# Synthesis, crystal structure and thermal properties of poly[[μ-1,2-bis­(pyridin-4-yl)ethene-κ^2^ *N*:*N*′-μ-bromido-copper(I)] 1,2-bis­(pyridin-4-yl)ethene 0.25-solvate]

**DOI:** 10.1107/S205698902300885X

**Published:** 2023-10-19

**Authors:** Christian Näther, Asmus Müller-Meinhard, Inke Jess

**Affiliations:** a Institut für Anorganische Chemie, Universität Kiel, Max-Eyth.-Str. 2, 24118 Kiel, Germany; University of Aberdeen, United Kingdom

**Keywords:** crystal structure, synthesis, thermal properties, copper(I), 1,2-bis­(pyridin-4-yl)ethene

## Abstract

The reaction of copper(I) bromide with 1,2-bis­(pyridin-4-yl)ethene in aceto­nitrile leads to the formation of the title compound, {[CuBr(C_12_H_10_N_2_)]·0.25C_12_H_10_N_2_}_*n*_ or CuBr(4-bpe)·0.25(4-bpe) [4-bpe = 1,2-bis­(pyridin-4-yl)ethene]. The asymmetric unit consists of one copper(I) cation and one bromide anion in general positions as well as two crystallographically independent half 4-bpe ligands and a quarter of a disordered 4-bpe solvate mol­ecule that are completed by centers of inversion. The copper(I) cations are tetra­hededrally coordinated as CuBr_2_N_2_ and linked by pairs of μ-1,1-bridging bromide anions into centrosymmetric dinuclear units that are further connected into layers by the 4-bpe coligands. Between the layers, inter­layer C—H⋯Br hydrogen bonding is observed. The layers are arranged in such a way that cavities are formed in which the disordered 4-bpe solvate mol­ecules are located. Powder X-ray (PXRD) investigations reveal that a pure sample has been obtained. Thermogravimetric (TG) and differential thermoanalysis (DTA) measurements show two mass losses that are accompanied by endothermic events in the DTA curve. The first mass loss correspond to the removal of 0.75 4-bpe mol­ecules, leading to the formation of (CuBr)_2_(4-bpe), already reported in the literature as proven by PXRD.

## Abstract 2

In the crystal structure of the title compound, the copper(I) cations are tetra­hededrally coordinated and linked by pairs of bromide anions into dinuclear units that are further connected into layers by the 1,2-bis­(pyridin-4-yl)ethene (4-bpe) coligands. The layers are stacked so that cavities are formed in which disordered 4-bpe mol­ecules are embedded.

## 1. Chemical context

Coordination polymers based on copper(I) halides show a large structural variability and are of inter­est, for example, regarding their luminescence behavior (Jess *et al.*, 2007 ▸; Peng *et al.*, 2010 ▸; Gibbons *et al.*, 2017 ▸; Jia *et al.*, 2018 ▸; Nitsch *et al.*, 2015 ▸; Mensah *et al.*, 2021 ▸). They consist of Cu*X* substructures including monomeric and dimeric units, chains, double chains and layers, which can be further connected into one-, two- and three-dimensional networks if bridging coligands are present (Peng *et al.*, 2010 ▸; Näther *et al.*, 2007 ▸; Kromp *et al.*, 2003 ▸). For a pairing of a particular copper(I) halide and coligand, frequently two or more compounds with a different ratio between the copper(I) halide and the coligand are found.

In previous investigations we have found that the coligand-rich compounds usually lose their coligands stepwise, which lead to the irreversible formation of ligand-deficient inter­mediates that are obtained in qu­anti­tative yield (Näther & Jess, 2004 ▸; Näther *et al.*, 2002 ▸). In the course of this reaction, compounds with more condensed Cu*X* substructures are formed. This is the case, *e.g.*, for coordination compounds based on pyrazine and 4,4′-bi­pyridine. With pyrazine, one compound with the composition CuCl(pyrazine) is known in which the copper(I) cations are linked by the chloride anions into chains, which are further connected into layers by the pyrazine ligands (Moreno *et al.*, 1995 ▸). Upon heating, half of the pyrazine ligands are removed, leading to a compound with the composition (CuCl)_*2*_(pyrazine), in which the Cu^I^ cations are linked by μ-1,1 bridging chloride anions into double chains, which are further connected into layers by the coligands (Kawata *et al.*, 1998 ▸; Näther *et al.*, 2001 ▸). 4,4′-Bi­pyridine compounds with the composition Cu*X*(4,4′-bi­pyridine) (*X* = Cl, Br, I) have been reported in which the copper cations are connected into (Cu*X*)_2_ dimeric units, which are further linked into layers by the 4,4′-bi­pyridine ligands (Yaghi & Li, 1995 ▸; Batten *et al.*, 1999 ▸; Lu *et al.*, 1999 ▸). Thermogravimetric experiments prove that the coligands are removed in a stepwise fashion leading to compounds with the composition (Cu*X*)_2_(4,4′-bi­pyridine) (*X* = Cl, Br, I), in which the Cu^I^ cations are linked into double chains, which are further connected into layers by bridging 4,4′-bi­pyridine ligands (Yaghi & Li, 1995 ▸; Näther & Jess, 2001 ▸).

A further bridging coligand is 1,2-bis­(pyridin-4-yl)ethene, for which some compounds have already been reported in the literature (see *Database survey*). These includes three ligand-deficient compounds with the composition (Cu*X*)_2_(4-bpe) (*X* = Cl, Br, I) in which the copper(I) cations are linked by the halide anions into chains, which are further connected into layers by the 4-bpe ligand (Li *et al.*, 2006 ▸; Yang & Li, 2006 ▸; Chen *et al.*, 2008 ▸; Wang, 2016 ▸; Shen & Lush, 2010 ▸; Blake *et al.*, 1999 ▸; Neal *et al.*, 2019 ▸). With CuI, a ligand-rich compound with the composition CuI(4-bpe)·0.25 4-bpe has already been reported, which is not known for CuBr and CuI (Hoffman *et al.*, 2020 ▸). This compound consists of layers that are stacked in such a way that pores are formed, in which 4-bpe solvate mol­ecules are located. A very similar structure is found for (CuCl)_2_(4-bpe)·4H_2_O, but in this compound the pores are filled with water, instead of 4-bpe (Mohapatra & Maji, 2010 ▸). Based on these findings, one can assume that a similar compound might also exist with CuBr. Moreover, for such a compound it is highly likely that upon heating it will transform into the ligand-deficient compound (CuBr)_2_(4-bpe) already reported in the literature. Therefore, we reacted CuBr with 4-bpe in different solvents and from aceto­nitrile we obtained a new crystalline phase that was characterized by single-crystal X-ray diffraction and thermoanalytical measurements. 
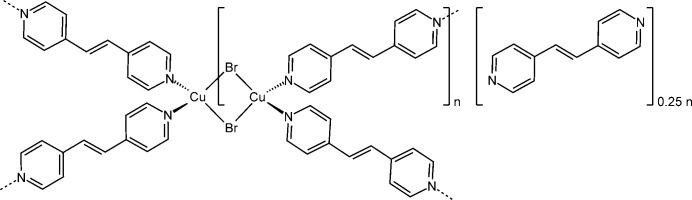


## 2. Structural commentary

**Figure 1:**
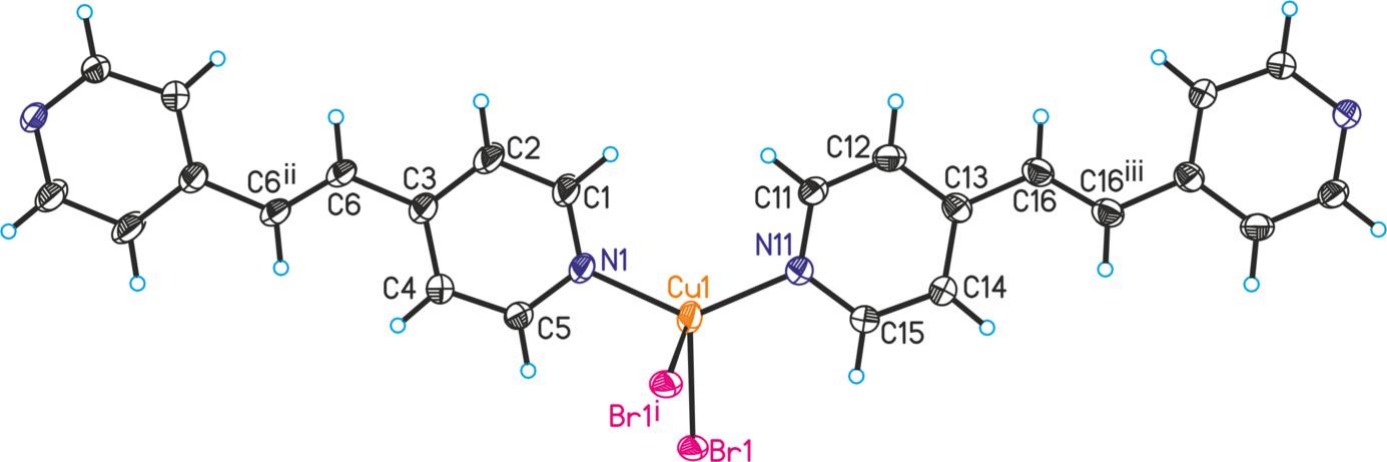
Crystal structure of the title compound with labeling and displacement ellipsoids drawn at the 50% probability level. Symmetry codes for the generation of equivalent atoms: (i) −*x*, −*y* + 2, −*z* + 1; (ii) −*x* − 1, −*y* + 2, −*z*; (iii) −*x* + 2, −*y* + 1, −*z* + 1.

**Figure 2:**
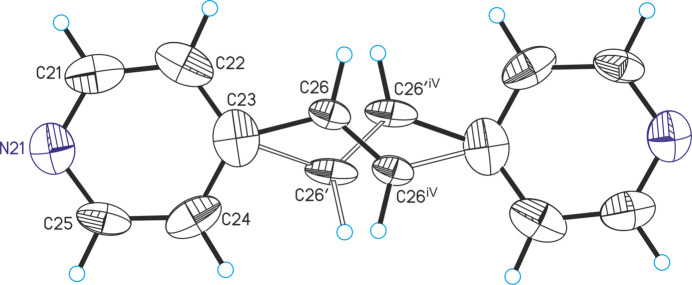
Crystal structure of the solvate 4-bpe mol­ecule with labeling and displacement ellipsoids drawn at the 50% probability level. Symmetry code for the generation of equivalent atoms: (iv) −*x* + 2, −*y* + 1, −*z*.

**Figure 3:**
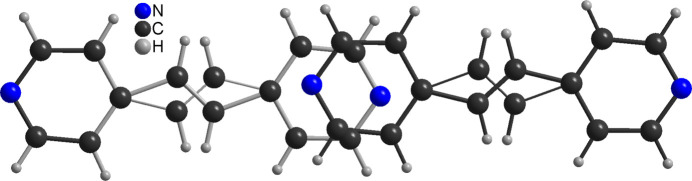
Crystal structure of the title compound showing the disorder of the solvate 4-bpe mol­ecule.

The title compound is isotypic to CuI(4-bpe)·0.25 4-bpe already reported in the literature (Hoffman *et al.*, 2020 ▸). Its asymmetric unit consists of one Cu^I^ cation and one bromide anion in general positions as well as two crystallographically independent half 4-bpe ligands that are completed by inversion symmetry (Fig. 1 ▸). There is one quarter of an additional bpe solvate mol­ecule that is disordered around a center of inversion (Fig. 2 ▸ and see *Refinement* section). Because of the disorder, this ligand is not fully occupied and was refined using a split model (Fig. 3 ▸).

**Figure 4:**
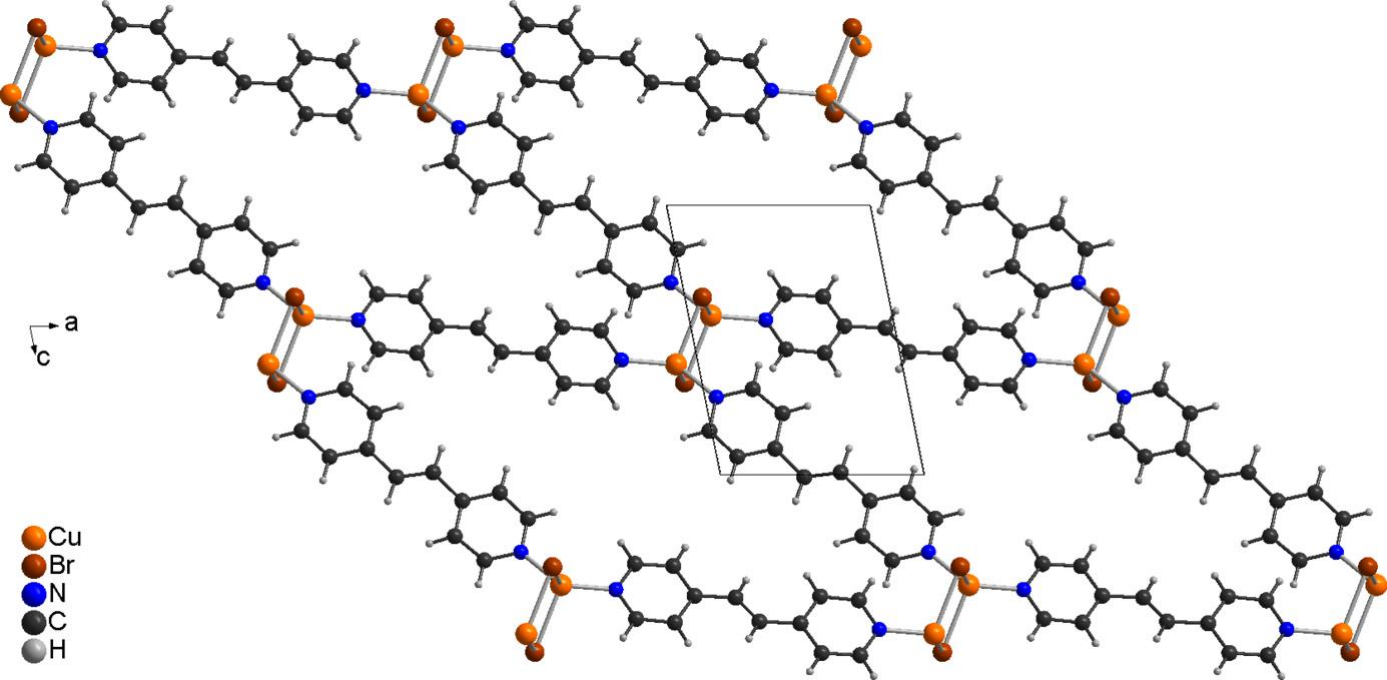
Crystal structure of the title compound with a view of one CuBr(4-bpe) layer along the crystallographic *b*-axis direction. The disordered 4-bpe solvate mol­ecule is not shown for clarity.

The copper(I) cations are tetra­hedrally coordinated by two symmetry-equivalent bromide anions and two N atoms of two crystallographically independent 4-bpe ligands (Fig. 1 ▸). From the bond lengths and angles (Table 1 ▸), it is apparent that the tetra­hedra are slightly distorted. Pairs of Cu^I^ cations are linked by two μ-1,1 bridging bromide anions into dimeric (CuBr)_2_ units that are located on centers of inversion and are further connected by the 4-bpe ligands into layers (Fig. 4 ▸).

## 3. Supra­molecular features

**Figure 5:**
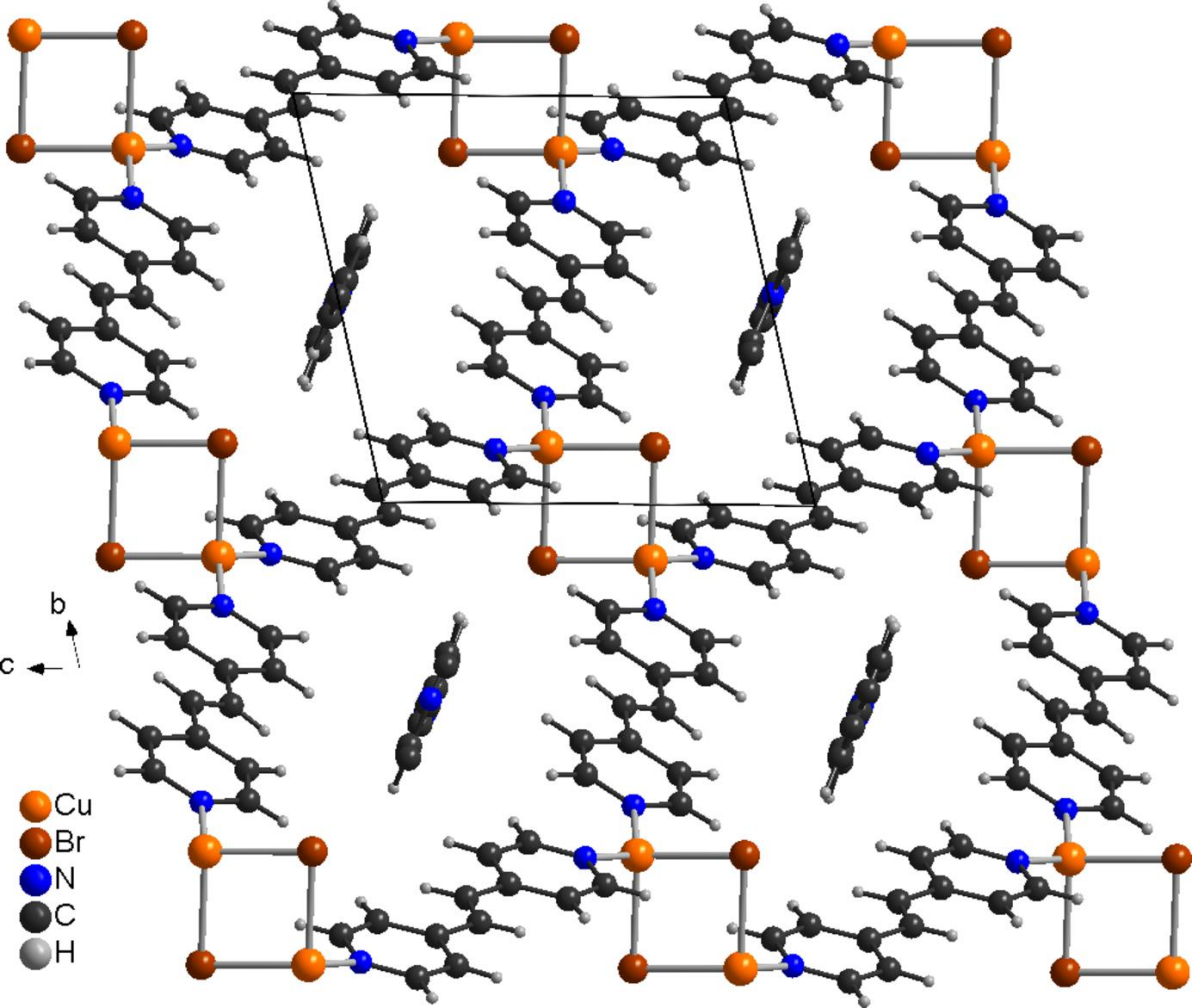
Crystal structure of the title compound with a view along the crystallographic *a*-axis direction, showing the pores in which the disordered solvate 4-bpe mol­ecules are embedded.

In the crystal structure of the title compound, the layers are arranged in such a way that cavities are formed, which proceed along the *a*-axis direction, in which the disordered 4-bpe solvate mol­ecules are embedded (Fig. 5 ▸). The layers are connected *via* inter­molecular C—H⋯Br hydrogen bonding (Table 2 ▸). The C—H⋯Br angle is close to linearity, indicating that this is a significant inter­action. There are additional C—H⋯Br inter­actions, between the C—H groupings of the solvate 4-bpe ligands and the bromide ions (Table 2 ▸).

## 4. Database survey

A search in the CSD database (version 5.43, last update November 2023; Groom *et al.*, 2016 ▸) using *ConQuest* (Bruno *et al.*, 2002 ▸) revealed that several compounds with copper(I) halides and 4-bpe as a coligand have been reported. These include three compounds with the composition (Cu*X*)_2_(4-bpe) with *X* = Cl (CSD refcode WEHVIP, Li *et al.*, 2006 ▸; WEHVIP01, Yang *et al.*, 2006 ▸; WEHVIP02, Chen *et al.*, 2008 ▸; WEHVIP03, Wang, 2016 ▸), Br (SUXSUA; Shen & Lush, 2010 ▸), I (HUJHID; Blake *et al.*, 1999 ▸; HUJHID01, Neal *et al.*, 2019 ▸). In all these compounds, the copper(I) cations are tetra­hedrally coordinated by three bromide anions and one 4-bpe ligand. The copper(I) cations are linked by the three μ-1,1,1 bridging halide anions into chains that are further linked into layers by the 4-bpe coligands. The chloride and iodide compounds are isotypic, which is not the case for the bromide compound. There is one compound of the composition (CuI)(4-bpe)·0.25 4-bpe that is isotypic to the title compound (TUYRAJ; Hoffman *et al.*, 2020 ▸). Another compound of the composition (CuCl)_2_(4-bpe)·4H_2_O has similar unit-cell parameters as well as the same space group, which indicates that this compound may also be isotypic to the title compound (HUTXIE; Mohapatra & Maji, 2010 ▸).

There are further compounds that additionally contain tri­phenyl­phosphane as ligand, such as (Cu*X*)_2_(4-bpe)(tri­phenyl­phosphane)_2_ with *X* = I (NAZTEQ; Sugimoto *et al.*, 2018 ▸), Br (SIPYEW; Yu *et al.*, 2007 ▸). One additional compound with the composition (CuCl)_2_(4-bpe)(tri­phenyl­phosphan)_2_·2 CH_2_Cl_2_ contains solvate mol­ecules (SIPYIA; Yu *et al.*, 2007 ▸).

## 5. Thermoanalytical investigations

**Figure 6:**
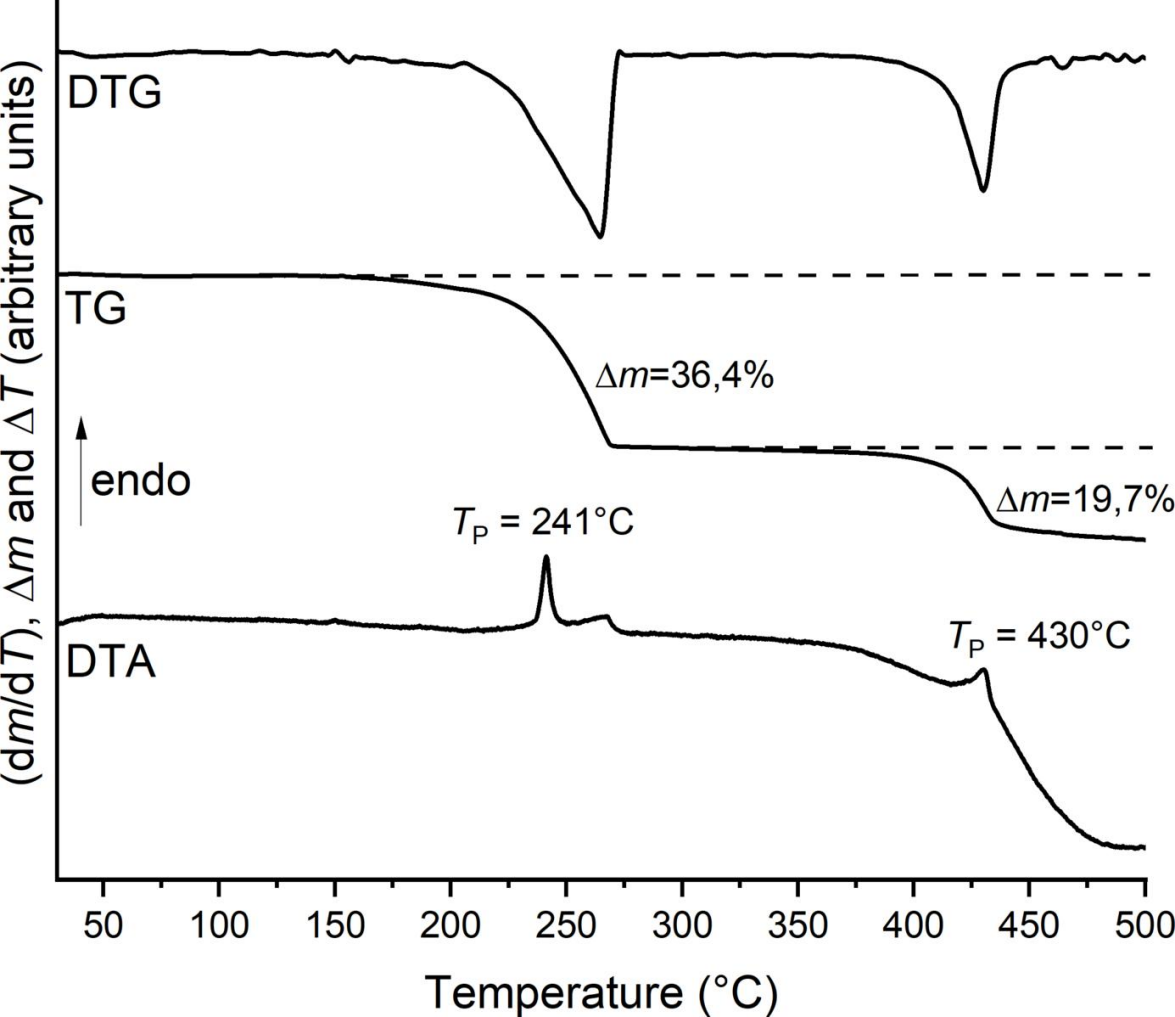
DTG, TG and DTA curve for the title compound, measured with a 4°C min^−1^ heating rate.

Comparison of the experimental powder pattern with that calculated from single-crystal data reveals that the title compound was obtained as a pure phase (Fig. S1). The title compound was characterized for its thermal properties by simultaneous thermogravimetry and differential thermoanalysis (TG–DTA). Upon heating, two mass losses are observed in the TG curve that are accompanied by endothermic events in the DTA curve (Fig. 6 ▸). From the first derivative of the TG curve (DTG curve), it is obvious that both mass losses are well resolved (Fig. 6 ▸). The first mass loss of 36.4% is in good agreement with that calculated for the removal of 0.75 4-bpe ligands (Δ*m*_calc._= 36.8%), whereas the second mass loss of 19.7% is much lower than that expected for the loss of the remaining 4-bpe ligands (Δ*m*_calc._= 24.5%), indicating that in this step the coligands are not completely removed. However, the first observation indicates that after the first mass loss a compound with the composition (CuBr)_2_(4-bpe) has been formed. To prove this assumption, a second TG measurement was performed, in which the residue formed after the first mass loss was isolated and investigated by PXRD. Comparison of the experimental pattern with that calculated for (CuBr)_2_(4-bpe) reported in the literature (Shen *et al.*, 2010 ▸) proves that this compound was obtained (Fig. S2).

## 6. Synthesis and crystallization

CuBr was purchased from Riedel de Haën. 4-bpe was purchased from Sigma-Aldrich. A microcrystalline powder was obtained by the reaction of 0.5 mmol CuBr (71.75 mg) and 1.0 mmol 4-bpe (182.2 mg) in 3 ml of MeCN. The mixture was stirred for 4 d at room temperature and filtered off. Crystals suitable for single-crystal X-ray diffraction were obtained under hydro­thermal conditions for 4 d at 403 K using 0.5 mmol of CuBr (71.75 mg), 2.0 mmol of 4-bpe (364.4 mg) in 3 ml of MeCN as a solvent. An IR spectrum of the title compound can be found in Fig.  S3.

**Experimental details**

The XRPD measurements were performed with a Stoe Transmission Powder Diffraction System (STADI P) equipped with a MYTHEN 1K detector and a Johansson-type Ge(111) monochromator using Cu *K*α_1_ radiation (λ = 1.540598 Å). The IR spectra were measured using an ATI Mattson Genesis Series FTIR Spectrometer, control software: *WINFIRST*, from ATI Mattson. Thermogravimetry and differential thermoanalysis (TG–DTA) measurements were performed in a dynamic nitro­gen atmosphere in Al_2_O_3_ crucibles using a STA-PT 1000 thermobalance from Linseis. The instrument was calibrated using standard reference materials.

## 7. Refinement

Crystal data, data collection and structure refinement details are summarized in Table 3 ▸. The C-bound H atoms were positioned with idealized geometry and were refined isotropically with *U*_ĩso_(H) = 1.2*U*_eq_(C) using a riding model. The solvate 4-bpe mol­ecule is disordered around a center of inversion. Therefore, it was refined using a split model with restraints for the geometry (SAME) and half occupancy for all atoms.

## Supplementary Material

Crystal structure: contains datablock(s) I. DOI: 10.1107/S205698902300885X/hb8078sup1.cif

Structure factors: contains datablock(s) I. DOI: 10.1107/S205698902300885X/hb8078Isup2.hkl

Experimental (top) and calculated powder pattern (bottom) of the title compound. DOI: 10.1107/S205698902300885X/hb8078sup3.png

Experimental powder pattern of the residue obtained after the first mass loss in a TG measurement of the title compound (top) and calculated powder pattern for (CuBR)2(4-bpe) retrieved from literature. DOI: 10.1107/S205698902300885X/hb8078sup4.png

IR spectrum of the title compound. The values of the most prominent vibrations are given. DOI: 10.1107/S205698902300885X/hb8078sup5.png

CCDC reference: 2300129

Additional supporting information:  crystallographic information; 3D view; checkCIF report

## Figures and Tables

**Table 1:** Selected geometric parameters (Å, °)

Cu1—Br1	2.5441 (5)	Cu1—N1	1.988 (2)
Cu1—Br1^i^	2.6424 (5)	Cu1—N11	1.979 (2)
			
Br1—Cu1—Br1^i^	96.351 (16)	N11—Cu1—Br1	107.21 (6)
N1—Cu1—Br1^i^	99.06 (7)	N11—Cu1—N1	131.18 (9)
N1—Cu1—Br1	108.16 (6)	Cu1—Br1—Cu1^i^	83.649 (16)
N11—Cu1—Br1^i^	109.28 (6)		

Symmetry code: (i) 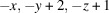 .

**Table 2:** Hydrogen-bond geometry (Å, °)

*D*—H⋯*A*	*D*—H	H⋯*A*	*D*⋯*A*	*D*—H⋯*A*
C4—H4⋯Br1^ii^	0.95	2.97	3.919 (3)	175
C5—H5⋯Br1	0.95	3.12	3.759 (2)	126
C24—H24⋯Br1^iii^	0.95	2.93	3.816 (7)	156
C26—H26⋯Br1^iv^	0.95	2.96	3.861 (12)	159
C26′—H26′⋯Br1^iii^	0.95	2.87	3.751 (17)	154

Symmetry codes: (ii) 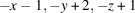 ; (iii) 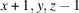 ; (iv) 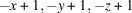 .

**Table 3:** Experimental details

Crystal data	
Chemical formula	[CuBr(C_12_H_10_N_2_)]·0.25C_12_H_10_N_2_
*M*_r_	370.72
Crystal system, space group	Triclinic, *P* 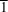
Temperature (K)	100
*a*, *b*, *c* (Å)	7.7421 (2), 10.1612 (2), 10.1749 (3)
α, β, γ (°)	72.143 (2), 73.252 (3), 68.004 (2)
*V* (Å^3^)	692.68 (4)
*Z*	2
Radiation type	Cu *K*α
μ (mm^−1^)	5.50
Crystal size (mm)	0.16 × 0.10 × 0.08

Data collection	
Diffractometer	XtaLAB Synergy, Dualflex, HyPix
Absorption correction	Multi-scan (*CrysAlis PRO*; Rigaku OD, 2023 ▸)
*T*_min_, *T*_max_	0.686, 1.000
No. of measured, independent and observed [*I* > 2σ(*I*)] reflections	15373, 2913, 2872
*R*_int_	0.023
(sin θ/λ)_max_ (Å^−1^)	0.639

Refinement	
*R*[*F* ^2^ > 2σ(*F* ^2^)], *wR*(*F* ^2^), *S*	0.029, 0.076, 1.09
No. of reflections	2913
No. of parameters	217
No. of restraints	16
H-atom treatment	H-atom parameters constrained
Δρ_max_, Δρ_min_ (e Å^−3^)	0.60, −0.62

Computer programs: *CrysAlis PRO* (Rigaku OD, 2023 ▸), *SHELXT2014/5* (Sheldrick, 2015*a* ▸), *SHELXL2016/6* (Sheldrick, 2015*b* ▸), *DIAMOND* (Brandenburg & Putz, 1999 ▸) and *publCIF* (Westrip, 2010 ▸).

## supplementary crystallographic information

### Crystal data

**Table:**

[CuBr(C_12_H_10_N_2_)]·0.25C_12_H_10_N_2_	*Z* = 2
*M**_r_* = 370.72	*F*(000) = 368
Triclinic, *P*1	*D*_x_ = 1.780 Mg m^−^^3^
*a* = 7.7421 (2) Å	Cu *K*α radiation, λ = 1.54184 Å
*b* = 10.1612 (2) Å	Cell parameters from 12191 reflections
*c* = 10.1749 (3) Å	θ = 4.7–77.9°
α = 72.143 (2)°	µ = 5.50 mm^−^^1^
β = 73.252 (3)°	*T* = 100 K
γ = 68.004 (2)°	Block, red
*V* = 692.68 (4) Å^3^	0.16 × 0.10 × 0.08 mm

### Data collection

**Table:**

XtaLAB Synergy, Dualflex, HyPix diffractometer	2913 independent reflections
Radiation source: micro-focus sealed X-ray tube, PhotonJet (Cu) X-ray Source	2872 reflections with *I* > 2σ(*I*)
Mirror monochromator	*R*_int_ = 0.023
Detector resolution: 10.0000 pixels mm^-1^	θ_max_ = 80.1°, θ_min_ = 4.7°
ω scans	*h* = −9→9
Absorption correction: multi-scan (CrysalisPro; Rigaku OD, 2023)	*k* = −12→12
*T*_min_ = 0.686, *T*_max_ = 1.000	*l* = −12→10
15373 measured reflections	

### Refinement

**Table:**

Refinement on *F*^2^	Primary atom site location: dual
Least-squares matrix: full	Hydrogen site location: inferred from neighbouring sites
*R*[*F*^2^ > 2σ(*F*^2^)] = 0.029	H-atom parameters constrained
*wR*(*F*^2^) = 0.076	*w* = 1/[σ^2^(*F*_o_^2^) + (0.0325*P*)^2^ + 1.2491*P*] where *P* = (*F*_o_^2^ + 2*F*_c_^2^)/3
*S* = 1.09	(Δ/σ)_max_ = 0.001
2913 reflections	Δρ_max_ = 0.60 e Å^−^^3^
217 parameters	Δρ_min_ = −0.62 e Å^−^^3^
16 restraints	

### Special details

**Table:**

Geometry. All esds (except the esd in the dihedral angle between two l.s. planes) are estimated using the full covariance matrix. The cell esds are taken into account individually in the estimation of esds in distances, angles and torsion angles; correlations between esds in cell parameters are only used when they are defined by crystal symmetry. An approximate (isotropic) treatment of cell esds is used for estimating esds involving l.s. planes.

### Fractional atomic coordinates and isotropic or equivalent isotropic displacement parameters (Å^2^)

**Table:**

	*x*	*y*	*z*	*U*_iso_*/*U*_eq_	Occ. (<1)
Cu1	0.10927 (5)	0.86193 (5)	0.40925 (4)	0.03213 (12)	
Br1	−0.08547 (4)	0.86001 (3)	0.65801 (3)	0.02641 (9)	
N1	−0.0555 (3)	0.8695 (3)	0.2873 (2)	0.0281 (5)	
C1	0.0113 (5)	0.8330 (6)	0.1631 (4)	0.0727 (16)	
H1	0.143170	0.783182	0.139491	0.087*	
C2	−0.1003 (5)	0.8634 (7)	0.0665 (4)	0.0795 (17)	
H2	−0.044548	0.834750	−0.020707	0.095*	
C3	−0.2928 (4)	0.9353 (3)	0.0968 (3)	0.0311 (6)	
C4	−0.3642 (4)	0.9700 (3)	0.2276 (3)	0.0253 (5)	
H4	−0.496090	1.017358	0.255058	0.030*	
C5	−0.2425 (4)	0.9356 (3)	0.3180 (3)	0.0244 (5)	
H5	−0.294996	0.960275	0.407184	0.029*	
C6	−0.4080 (4)	0.9702 (3)	−0.0088 (3)	0.0329 (6)	
H6	−0.342795	0.947368	−0.097537	0.039*	
N11	0.3736 (3)	0.7409 (2)	0.4259 (2)	0.0234 (4)	
C11	0.4923 (4)	0.6648 (3)	0.3305 (3)	0.0269 (5)	
H11	0.445587	0.666766	0.252948	0.032*	
C12	0.6785 (4)	0.5838 (3)	0.3389 (3)	0.0284 (5)	
H12	0.755308	0.529594	0.269785	0.034*	
C13	0.7539 (3)	0.5816 (3)	0.4487 (3)	0.0242 (5)	
C14	0.6307 (4)	0.6606 (3)	0.5484 (3)	0.0261 (5)	
H14	0.674801	0.662599	0.625570	0.031*	
C15	0.4447 (3)	0.7359 (3)	0.5344 (3)	0.0258 (5)	
H15	0.362545	0.786845	0.604744	0.031*	
C16	0.9534 (4)	0.4976 (3)	0.4555 (3)	0.0266 (5)	
H16	1.020879	0.435330	0.391822	0.032*	
N21	0.3823 (9)	0.5175 (8)	−0.0098 (6)	0.0499 (14)	0.5
C21	0.493 (2)	0.4013 (17)	0.062 (3)	0.051 (4)	0.5
H21	0.443905	0.323171	0.111592	0.061*	0.5
C22	0.6762 (13)	0.3836 (8)	0.0708 (7)	0.0514 (19)	0.5
H22	0.748525	0.295564	0.123189	0.062*	0.5
C23	0.7524 (10)	0.4965 (8)	0.0020 (7)	0.0494 (17)	0.5
C24	0.6363 (12)	0.6207 (7)	−0.0705 (7)	0.0495 (18)	0.5
H24	0.680607	0.701567	−0.119425	0.059*	0.5
C25	0.455 (2)	0.6270 (18)	−0.071 (3)	0.044 (3)	0.5
H25	0.377125	0.715220	−0.119266	0.052*	0.5
C26	0.9471 (15)	0.4575 (12)	0.0265 (10)	0.0280 (18)	0.3
H26	0.997754	0.365000	0.084446	0.034*	0.3
C26'	0.930 (3)	0.546 (2)	−0.0285 (15)	0.038 (4)	0.2
H26'	0.934250	0.638394	−0.085763	0.045*	0.2

### Atomic displacement parameters (Å^2^)

**Table:**

	*U*^11^	*U*^22^	*U*^33^	*U*^12^	*U*^13^	*U*^23^
Cu1	0.0218 (2)	0.0507 (3)	0.0213 (2)	−0.00529 (17)	−0.00852 (15)	−0.00798 (17)
Br1	0.02694 (14)	0.02953 (15)	0.01730 (14)	−0.00214 (10)	−0.00306 (10)	−0.00738 (10)
N1	0.0224 (10)	0.0430 (13)	0.0183 (11)	−0.0073 (9)	−0.0066 (8)	−0.0076 (9)
C1	0.0211 (14)	0.156 (5)	0.0325 (19)	0.005 (2)	−0.0076 (13)	−0.049 (2)
C2	0.0277 (16)	0.170 (5)	0.0333 (19)	0.006 (2)	−0.0089 (14)	−0.055 (3)
C3	0.0258 (13)	0.0462 (16)	0.0215 (13)	−0.0083 (11)	−0.0067 (10)	−0.0096 (11)
C4	0.0238 (12)	0.0261 (12)	0.0248 (13)	−0.0044 (9)	−0.0073 (9)	−0.0062 (10)
C5	0.0269 (12)	0.0240 (11)	0.0227 (12)	−0.0050 (9)	−0.0071 (9)	−0.0075 (9)
C6	0.0273 (13)	0.0525 (17)	0.0200 (13)	−0.0077 (11)	−0.0053 (10)	−0.0153 (12)
N11	0.0221 (10)	0.0265 (10)	0.0212 (10)	−0.0063 (8)	−0.0053 (8)	−0.0057 (8)
C11	0.0295 (13)	0.0280 (12)	0.0238 (13)	−0.0040 (10)	−0.0093 (10)	−0.0090 (10)
C12	0.0298 (13)	0.0290 (13)	0.0244 (13)	−0.0044 (10)	−0.0035 (10)	−0.0110 (10)
C13	0.0233 (12)	0.0207 (11)	0.0269 (13)	−0.0048 (9)	−0.0047 (9)	−0.0056 (9)
C14	0.0241 (12)	0.0304 (12)	0.0257 (13)	−0.0061 (10)	−0.0079 (10)	−0.0092 (10)
C15	0.0223 (11)	0.0308 (13)	0.0243 (13)	−0.0049 (10)	−0.0041 (9)	−0.0109 (10)
C16	0.0245 (12)	0.0240 (12)	0.0295 (14)	−0.0038 (9)	−0.0031 (10)	−0.0102 (10)
N21	0.048 (3)	0.067 (5)	0.037 (3)	−0.017 (3)	−0.002 (3)	−0.020 (3)
C21	0.068 (10)	0.035 (7)	0.035 (8)	−0.011 (8)	0.011 (7)	−0.014 (6)
C22	0.065 (5)	0.049 (4)	0.024 (3)	0.006 (4)	−0.009 (3)	−0.015 (3)
C23	0.046 (4)	0.073 (5)	0.035 (4)	−0.012 (3)	0.005 (3)	−0.039 (4)
C24	0.071 (5)	0.046 (4)	0.031 (3)	−0.032 (4)	0.016 (3)	−0.016 (3)
C25	0.054 (8)	0.032 (6)	0.025 (4)	0.000 (6)	−0.003 (6)	−0.001 (5)
C26	0.036 (6)	0.026 (6)	0.014 (4)	−0.003 (4)	−0.003 (4)	−0.003 (4)
C26'	0.061 (13)	0.030 (9)	0.008 (6)	−0.009 (8)	−0.006 (7)	0.007 (6)

### Geometric parameters (Å, º)

**Table:**

Cu1—Br1	2.5441 (5)	C14—C15	1.381 (3)
Cu1—Br1^i^	2.6424 (5)	C15—H15	0.9500
Cu1—N1	1.988 (2)	C16—C16^iii^	1.331 (5)
Cu1—N11	1.979 (2)	C16—H16	0.9500
N1—C1	1.330 (4)	N21—N21^iv^	1.781 (13)
N1—C5	1.336 (3)	N21—C21	1.319 (13)
C1—H1	0.9500	N21—C21^iv^	1.386 (18)
C1—C2	1.382 (4)	N21—C22^iv^	1.019 (9)
C2—H2	0.9500	N21—C23^iv^	1.081 (9)
C2—C3	1.381 (4)	N21—C24^iv^	1.428 (10)
C3—C4	1.385 (4)	N21—C25^iv^	1.701 (16)
C3—C6	1.468 (4)	N21—C25	1.337 (15)
C4—H4	0.9500	C21—H21	0.9500
C4—C5	1.382 (3)	C21—C22	1.385 (13)
C5—H5	0.9500	C22—H22	0.9500
C6—C6^ii^	1.305 (5)	C22—C23	1.389 (11)
C6—H6	0.9500	C23—C24	1.381 (10)
N11—C11	1.341 (3)	C23—C26	1.484 (12)
N11—C15	1.349 (3)	C23—C26'	1.55 (2)
C11—H11	0.9500	C24—H24	0.9500
C11—C12	1.379 (4)	C24—C25	1.382 (14)
C12—H12	0.9500	C25—H25	0.9500
C12—C13	1.393 (4)	C26—C26^v^	1.30 (2)
C13—C14	1.397 (3)	C26—H26	0.9500
C13—C16	1.469 (3)	C26'—C26'^v^	1.29 (3)
C14—H14	0.9500	C26'—H26'	0.9500
			
Br1—Cu1—Br1^i^	96.351 (16)	C22^iv^—N21—N21^iv^	115.6 (9)
N1—Cu1—Br1^i^	99.06 (7)	C22^iv^—N21—C21	166.0 (9)
N1—Cu1—Br1	108.16 (6)	C22^iv^—N21—C21^iv^	68.3 (8)
N11—Cu1—Br1^i^	109.28 (6)	C22^iv^—N21—C23^iv^	82.7 (8)
N11—Cu1—Br1	107.21 (6)	C22^iv^—N21—C24^iv^	147.8 (9)
N11—Cu1—N1	131.18 (9)	C22^iv^—N21—C25^iv^	160.5 (10)
Cu1—Br1—Cu1^i^	83.649 (16)	C22^iv^—N21—C25	51.4 (8)
C1—N1—Cu1	123.56 (19)	C23^iv^—N21—N21^iv^	161.7 (9)
C1—N1—C5	116.2 (2)	C23^iv^—N21—C21	111.2 (9)
C5—N1—Cu1	119.41 (17)	C23^iv^—N21—C21^iv^	151.1 (10)
N1—C1—H1	118.1	C23^iv^—N21—C24^iv^	65.1 (6)
N1—C1—C2	123.7 (3)	C23^iv^—N21—C25^iv^	116.6 (9)
C2—C1—H1	118.1	C23^iv^—N21—C25	134.0 (10)
C1—C2—H2	120.0	C24^iv^—N21—N21^iv^	96.6 (6)
C3—C2—C1	120.0 (3)	C24^iv^—N21—C25^iv^	51.5 (6)
C3—C2—H2	120.0	C25—N21—N21^iv^	64.3 (7)
C2—C3—C4	116.5 (2)	C25^iv^—N21—N21^iv^	45.1 (6)
C2—C3—C6	119.2 (3)	C25—N21—C21^iv^	17.3 (9)
C4—C3—C6	124.3 (2)	C25—N21—C24^iv^	160.9 (9)
C3—C4—H4	120.2	C25—N21—C25^iv^	109.4 (8)
C5—C4—C3	119.7 (2)	N21—C21—H21	117.3
C5—C4—H4	120.2	N21—C21—C22	125.5 (12)
N1—C5—C4	123.8 (2)	C22—C21—H21	117.3
N1—C5—H5	118.1	C21—C22—H22	120.5
C4—C5—H5	118.1	C21—C22—C23	119.1 (10)
C3—C6—H6	117.2	C23—C22—H22	120.5
C6^ii^—C6—C3	125.7 (3)	N21^iv^—C23—C21^iv^	38.3 (6)
C6^ii^—C6—H6	117.2	N21^iv^—C23—C22	46.7 (6)
C11—N11—Cu1	123.12 (17)	N21^iv^—C23—C24	69.7 (7)
C11—N11—C15	116.7 (2)	N21^iv^—C23—C26	157.5 (9)
C15—N11—Cu1	120.17 (17)	N21^iv^—C23—C26'	168.7 (10)
N11—C11—H11	118.2	C22—C23—C21^iv^	85.0 (6)
N11—C11—C12	123.5 (2)	C22—C23—C26	110.8 (8)
C12—C11—H11	118.2	C22—C23—C26'	144.6 (9)
C11—C12—H12	120.0	C24—C23—C21^iv^	31.5 (6)
C11—C12—C13	120.0 (2)	C24—C23—C22	116.4 (7)
C13—C12—H12	120.0	C24—C23—C26	132.8 (8)
C12—C13—C14	116.7 (2)	C24—C23—C26'	99.0 (9)
C12—C13—C16	119.7 (2)	C26—C23—C21^iv^	164.2 (8)
C14—C13—C16	123.6 (2)	C23—C24—H21^iv^	164.4 (16)
C13—C14—H14	120.1	C23—C24—H24	120.1
C15—C14—C13	119.8 (2)	C23—C24—C25	119.7 (8)
C15—C14—H14	120.1	H24—C24—H21^iv^	74.4
N11—C15—C14	123.3 (2)	C25—C24—H21^iv^	46.1 (18)
N11—C15—H15	118.3	C25—C24—H24	120.1
C14—C15—H15	118.3	N21—C25—H21^iv^	158 (2)
C13—C16—H16	117.6	N21—C25—C24	124.6 (12)
C16^iii^—C16—C13	124.8 (3)	N21—C25—H25	117.7
C16^iii^—C16—H16	117.6	C24—C25—H21^iv^	34.3 (10)
C21—N21—N21^iv^	50.5 (8)	C24—C25—H25	117.7
C21^iv^—N21—N21^iv^	47.2 (6)	H25—C25—H21^iv^	83.9
C21—N21—C21^iv^	97.7 (9)	C23—C26—H26	118.0
C21^iv^—N21—C24^iv^	143.8 (8)	C26^v^—C26—C23	124.0 (14)
C21—N21—C24^iv^	46.2 (8)	C26^v^—C26—H26	118.0
C21—N21—C25	114.7 (10)	C23—C26'—H26'	121.9
C21^iv^—N21—C25^iv^	92.3 (8)	C26'^v^—C26'—C23	116 (2)
C21—N21—C25^iv^	5.9 (13)	C26'^v^—C26'—H26'	121.9

Symmetry codes: (i) −*x*, −*y*+2, −*z*+1; (ii) −*x*−1, −*y*+2, −*z*; (iii) −*x*+2, −*y*+1, −*z*+1; (iv) −*x*+1, −*y*+1, −*z*; (v) −*x*+2, −*y*+1, −*z*.

### Hydrogen-bond geometry (Å, º)

**Table:**

*D*—H···*A*	*D*—H	H···*A*	*D*···*A*	*D*—H···*A*
C4—H4···Br1^vi^	0.95	2.97	3.919 (3)	175
C5—H5···Br1	0.95	3.12	3.759 (2)	126
C24—H24···Br1^vii^	0.95	2.93	3.816 (7)	156
C26—H26···Br1^viii^	0.95	2.96	3.861 (12)	159
C26′—H26′···Br1^vii^	0.95	2.87	3.751 (17)	154

Symmetry codes: (vi) −*x*−1, −*y*+2, −*z*+1; (vii) *x*+1, *y*, *z*−1; (viii) −*x*+1, −*y*+1, −*z*+1.
