# Supplementary figures and images for: Synthesis, crystal structure and thermal properties of poly[[μ-1,2-bis­(pyridin-4-yl)ethene-κ2 N:N′-μ-bromido-copper(I)] 1,2-bis­(pyridin-4-yl)ethene 0.25-solvate]
Source: Acta Crystallogr E Crystallogr Commun. 2023 Oct 19;79(Pt 11):1028–32. doi: 10.1107/S205698902300885X (PMC10626962; doi:10.1107/S205698902300885X)

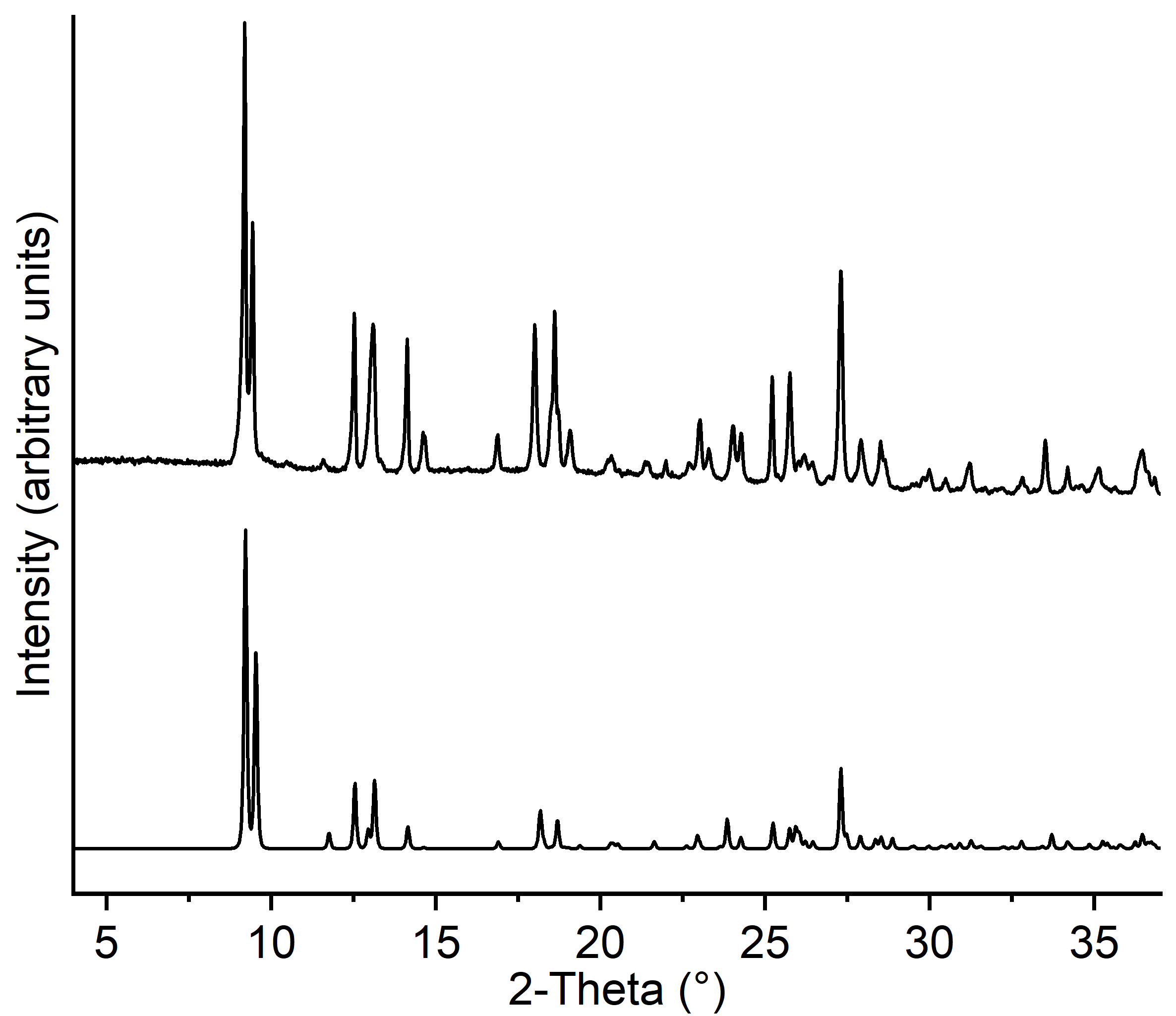

Supplement: Supplementary file 3 [file e-79-01028-sup3.png]

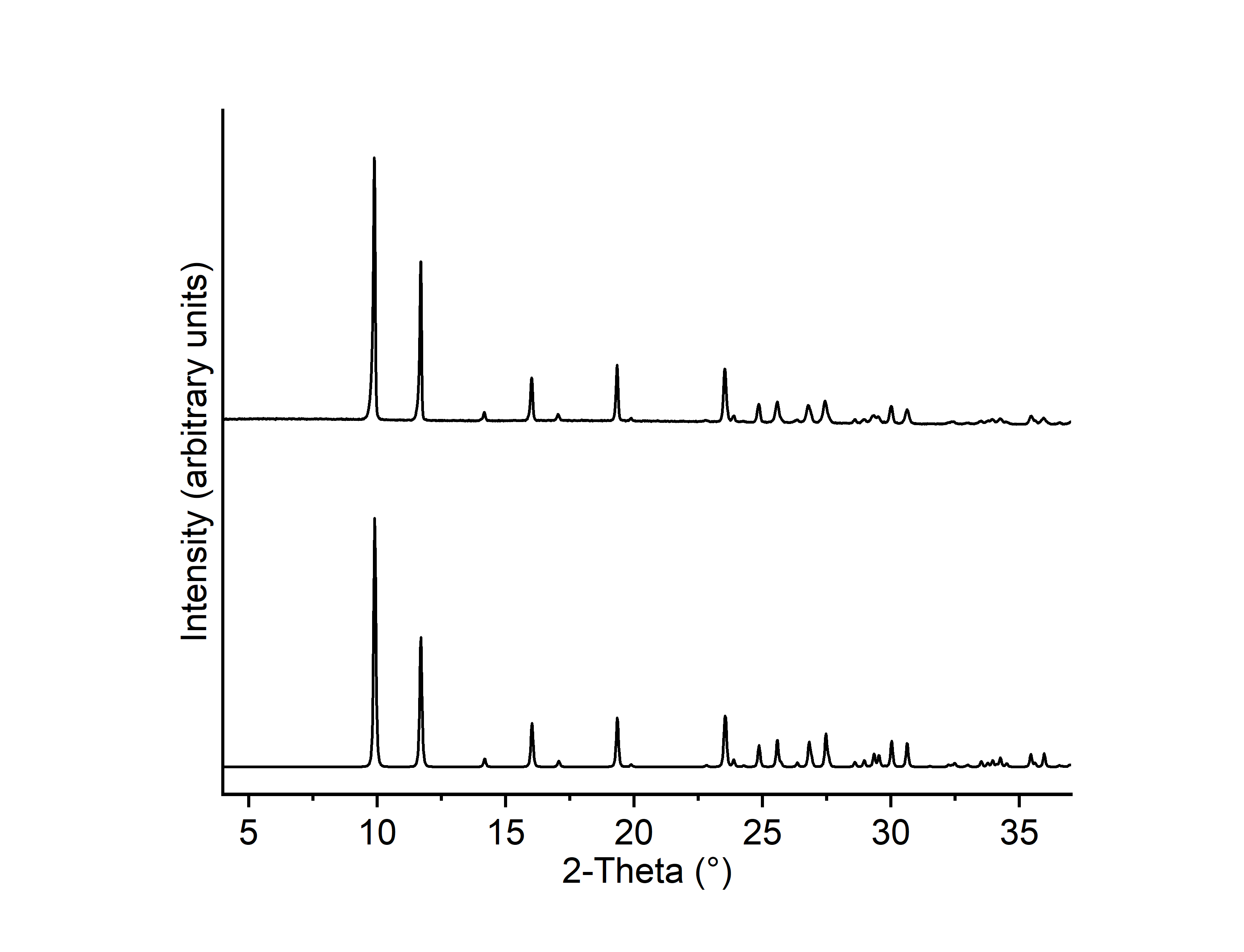

Supplement: Supplementary file 4 [file e-79-01028-sup4.png]

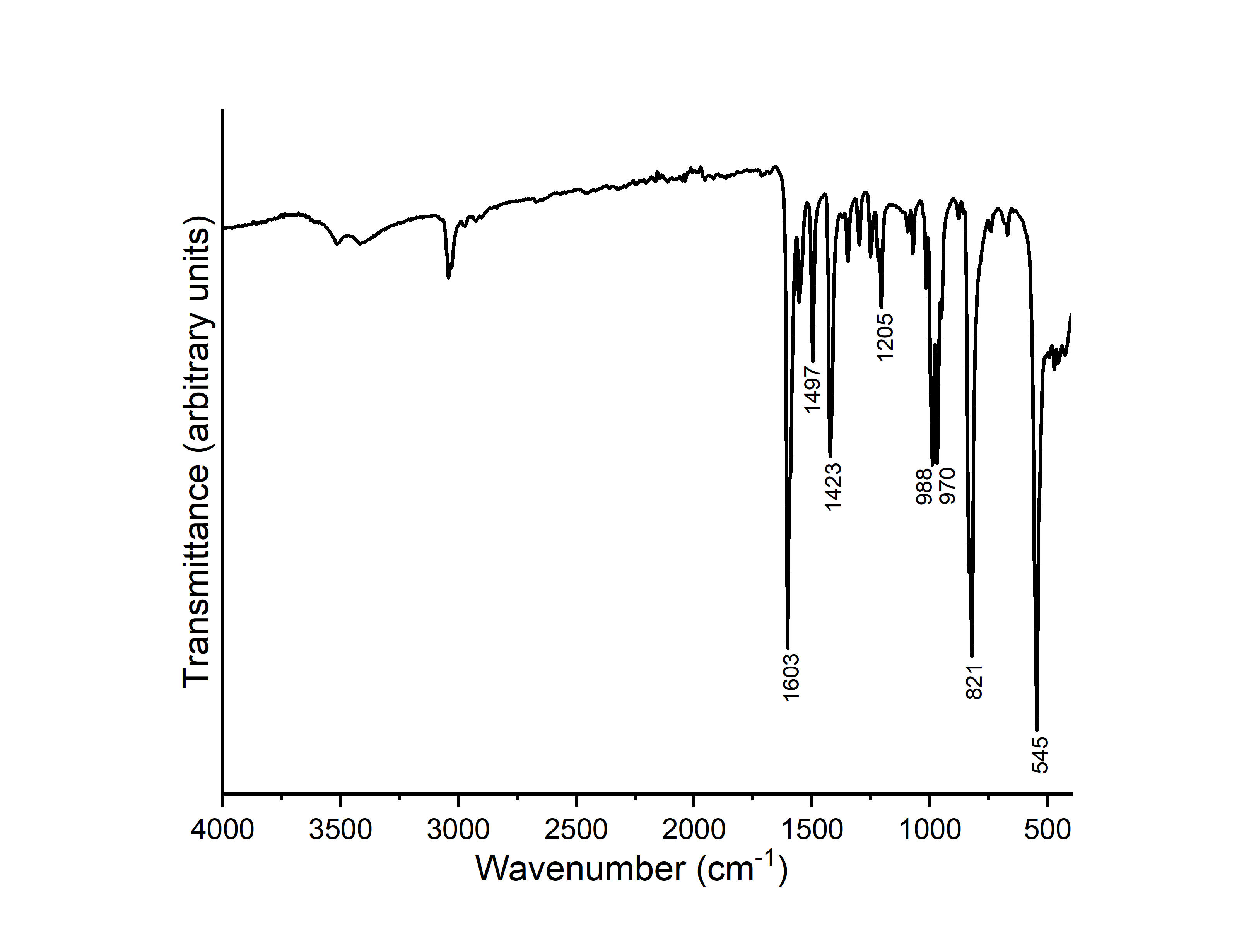

Supplement: Supplementary file 5 [file e-79-01028-sup5.png]
